# Gene expression profile analyze the molecular mechanism of CXCR7 regulating papillary thyroid carcinoma growth and metastasis

**DOI:** 10.1186/s13046-015-0132-y

**Published:** 2015-02-12

**Authors:** Hengwei Zhang, Xuyong Teng, Zhangyi Liu, Lei Zhang, Zhen Liu

**Affiliations:** Department General Surgery, Affiliated Shenjing Hospital, China Medical University, No.36 Sanhao Street, Shenyang, 110004 China

**Keywords:** Thyroid carcinoma, Chemokine receptor, CXCR7, Invasion, Metastasis, Gene microarray, Signal pathway

## Abstract

**Background:**

To detect genetic expression profile alterations after papillary thyroid carcinoma (PTC) cells transfected with chemokine receptor CXCR7 gene by gene microarray, and gain insights into molecular mechanisms of how CXCR7 regulating PTC growth and metastasis.

**Methods:**

The Human OneArray microarray was used for a complete genome-wide transcript profiling of CXCR7 transfected PTCs (K1-CXCR7 cells), defined as experimental group. Non CXCR7 transfected PTCs (K1 cells) were used as control group. Differential analysis for per gene was performed with a random variance model and t test, p values were adjusted to control the false discovery rate. Gene ontology (GO) on differentially expressed genes to identify the biological processes in modulating the progression of papillary thyroid carcinoma. Pathway analysis was used to evaluate the signaling pathway that differentially expressed genes were involved in. In addition, quantitative real-time polymerase chain reaction (q-PCR) and Western blot were used to verify the top differentially expression genes.

**Results:**

Comparative analysis revealed that the expression level of 1149 genes was changed in response to CXCR7 transfection. After unsupervised hierarchical clustering analysis, 270 differentially expressed genes were filtered, of them 156 genes were up-regulated whereas 114 genes were down-regulated in K1-CXCR7 cells. GO enrichment analysis revealed the differentially expressed genes were mainly involved in biopolymer metabolic process, signal transduction and protein metabolism. Pathway enrichment analysis revealed differentially expressed genes were mainly involved in ECM-receptor interaction, Focal adhesion, MAPK signaling pathway and Cytokine-cytokine receptor interaction pathway. More importantly, the expression level of genes closely associated with tumor growth and metastasis was altered significantly in K1-CXCR7 cells, including up-regulated genes FN1, COL1A1, COL4A1, PDGFRB, LTB, CXCL12, MMP-11, MT1-MMP and down-regulated genes ITGA7, and Notch-1.

**Conclusions:**

Gene expression profiling analysis of papillary thyroid carcinoma can further delineate the mechanistic insights on how CXCR7 regulating papillary thyroid carcinoma growth and metastasis. CXCR7 may regulate growth and metastasis of papillary thyroid carcinoma via the activation of PI3K/AKT pathway and its downstream NF-κB signaling, as well as the down-regulation of Notch signaling.

## Background

Thyroid carcinoma is the most common endocrine neoplasm, and its incidence has been explosively rising worldwide over the past few decades. Papillary thyroid carcinoma is the most common pathological type of thyroid carcinoma, accounting for at least 70–80% of thyroid carcinoma. Although PTC has a favorable prognosis, certain cases exhibit aggressive clinical characteristics, such as lymph node metastasis.

Chemokines and their receptors play a critical role in tumorigenesis, progression, and metastasis of tumor [1]. The chemokine receptor CXCR7 mediates cellular adhesion, migration, proliferation, and survival by binding its ligands stromal cell-derived factor-1(SDF-1) and Interferon-inducible T cell α-chemoattractant (I-TAC) [2,3]. In recent years, accumulating evidences had demonstrated that expression of CXCR7 played a critical role in tumor cell proliferation, angiogenesis, invasion, and metastasis [4-7]. In our previous study, we have demonstrated that CXCR7 and SDF-1 were over-expressed in PTC tissue compared with peritumoral nonmalignant tissue and thyroid benign lesion tissue, and the expressions of them were positively associated with lymph node metastasis [8]. In addition, we found that knockdown of CXCR7 in PTC cells suppressed cell proliferation, invasion, induced S phase arrest, and promoted apoptosis [9].

To further evaluate the signaling pathways involved in CXCR7 receptor regulated PTC progression, we used gene microarray to detect the altered gene expression in PTC cells transfected with CXCR7 and tried to gain insights into molecular mechanisms of how CXCR7 regulating PTC growth and metastasis.

## Methods

### Cell lines and culture conditions

The human papillary thyroid carcinoma cell line K1 was purchased from European Collection of Animal Cell Cultures. Stable human CXCR7 cDNA transfected cell line, K1-CXCR7, was established in our laboratory [9]. Both cells were cultured in Dulbecco’s Modified Eagle’s Medium: Ham’s F12:MCDB105 (Sigma-Aldrich, St. Louis, Missouri) containing 10% fetal calf serum (FCS; Sigma-Aldrich) and 2 mmol/L glutamine (Sigma-Aldrich). This study was approved by the Ethics Committee in the Affiliated Shengjing Hospital of China Medical University.

### RNA preparation and microarray analysis

The gene microarray analysis was carried out by Phalanx Biotech Group, which included RNA amplification, labeling of probe, hybridization, and data extraction. Briefly, total RNA was extracted from K1-CXCR7 cells as experimental group (O1,O2,O3) and K1 cells as control group (N1, N2, N3) using Trizol reagent (TaKaRa Bio Inc, Japan) according to the manufacturer’s instructions. RNA quantity and purity were assessed by using NanoDrop ND-1000 to measure OD260/280. RNA integrity was ascertained by using Agilent RNA 6000 Nano assay to determine RNA Integrity Number (RIN) values. Gene expression profiling was conducted with the Human OneArray® V6.1 microarray (OneArray, China Taiwan) containing 31741 human genome probes and 938 experimental control probes [10]. After hybridization, arrays were washed, scanned and then gene expression results were extracted by DNA Microarray Scanner G2565B (Agilent Technologies, United States) according to the manufacturer’s instructions. Raw fluorescence intensity values were normalized and log-transformed using GeneSpring GX 10 software (Agilent Technologies, United States).

### Quantitative real-time polymerase chain reaction

Total RNA was extracted using Trizol reagent (TaKaRa Bio Inc, Japan) according to the manufacturer’s instructions. Quantitative real-time polymerase chain reaction (q-PCR) analysis was performed on Lightcycler480 (Roche Applied Science) according to the manufacturer’s protocol. GAPDH was used as internal control to normalize mRNA levels. All the experiments were repeated three times. Primer sequences are listed in Table 1.

**Table 1:** **Primers used in this study**

**Genes**	**Primers**	**Length (bp)**
FN1	Forward:5′- GAGTGTGTGTGTCTTGGTAATGG-3′	108
	Reverse:5′- CCACGTTTCTCCGACCAC-3′	
COL1A1	Forward:5′- CCTGGATGCCATCAAAGTCT-3′	153
	Reverse:5′-AATCCATCGGTCATGCTCTC-3′	
COL4A1	Forward:5′- CTGGTCCAAGAGGATTTCCA-3′	193
	Reverse:5′-TCATTGCCTTGCACGTAGAG-3′	
PDGFR-β	Forward:5′- CTGGGCAAAAGGGACAAAGAG-3′	288
	Reverse:5′-CACTGGGCTGGGGACAATG-3′	
LTB	Forward:5′-CACAGGCCCAGCAAGGAC-3′	67
	Reverse:5′-GGGCTGAGATCTGTTTCTGG-3′	
CXCL12	Forward:5′- CCATGCCGATTCTTCGAAAG-3′	101
	Reverse:5′- TTCAGCCGGGCTACAATCTG-3′	
MMP-11	Forward:5′- AAGAGGTTCGTGCTTTCTGG -3′	72
	Reverse:5′- CCATGGGAACCGAAGGAT -3′	
MT1-MMP	Forward:5′-GAGCTCAGGGCAGTGGATAG-3′	172
	Reverse:5′-GGTAGCCCGGTTCTACCTTC-3′	
ITGA7	Forward:5′- GCTGTGAAGTCCCTGGAAGTGATT -3′	80
	Reverse:5′- GCATCTCGGAGCATCAAGTTCTT -3′	
Notch1	Forward:5′-CAATGTGGATGCCGCAGTTGTG-3′	124
	Reverse:5′-CAGCACCTTGGCGGTCTCGTA-3′	
GAPDH	Forward:5′- GCACCGTCAAGGCTGAGAAC-3′	138
	Reverse:5′-TGGTGAAGACGCCAGTGGA-3′	

### Western blotting

Cells were washed twice with ice-cold phosphate-buffered saline (PBS) and extracted according to protein extraction protocols. Protein concentrations were determined by the BCA Protein Assay Kit (Beyotime Biotechnology, China). Total protein samples (80 ug/lane) were electrophoresed on sodium dodecyl sulfate-polyacrylamide gel and then transferred to Polyvinylidene Fluoride membrane. After blocking with 5% non-fat dry milk for 2 h, membranes were incubated with primary antibodies overnight at 4°C. The membranes were incubated for 2 h at room temperature with secondary antibody. Antibodies used in this study included the following: rabbit polyclonal anti FN1 (1:400), anti-COL1A1 (1:200), anti-COL4A1 (1:500), anti-CXCL12 (1:400), anti-PDGFRB (1:200), anti-MMP-11 (1:200), anti-MTI-MMP (1:200), anti-ITGA7 (1:200, all from BOSTER, Wuhan, China), rabbit polyclonal anti-LTB (1:500, from Abcam, Cambridge, MA), and rabbit polyclonal anti-Notch1 (1:1000), GAPDH (1:10000, both from Proteintech Group Inc, Chicago, IL), and goat anti-rabbit IgG (1:2000; ZSGB-BIO, China) as secondary antibody. Binding was detected using the enhanced chemiluminescence reagents (Beyotime Biotechnology, China). The ratio between the integated optical density of interest proteins and GAPDH of the same sample was calculated as the relative content of protein detected. All experiments were performed in three times.

### Statistical analysis

Raw fluorescence intensity values were normalized and log-transformed. Fold change were calculated by Rosetta Resolver 7.2 with error model adjusted by Amersham Pairwise Ration Builder for signal comparison of sample. In bioinformatic analysis, differentially expressed genes were subjected to hierarchical clustering analysis, principal component analysis, Pathway analysis and Gene Ontology analysis. T-test was applied to analyze differences of measurement and categorical data. The SPSS 19.0 software (SPSS Inc., Chicago, IL) was used. All data were expressed as mean ± standard deviation (SD), and the p value <0.05 was considered statistically significant.

## Results

### Number of differentially expressed genes

**Figure 1:**
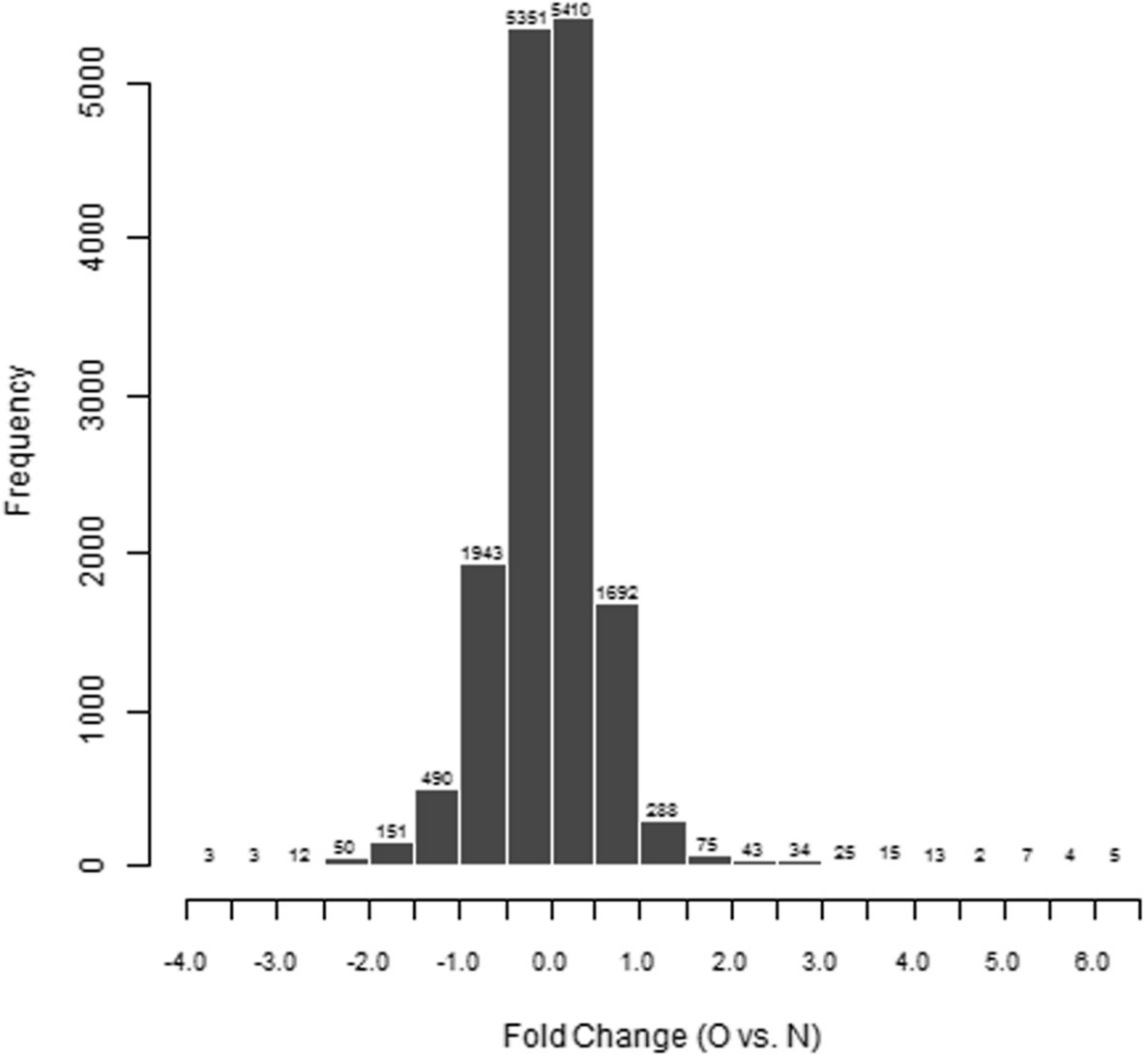
**Histograms with fold change between the experimental and control groups (O vs N).** Fold change represents logarithm of fluorescence signal intensity ratios for differentially expressed genes. And log2 ratios ≥ 1.0 or log2 ratios ≤ −1.0 means two Fold change. The histogram plot shows fold change distribution of all probes excluding control and flagged probes. |Fold change| ≥1 means genes differentially expressed.

**Figure 2:**
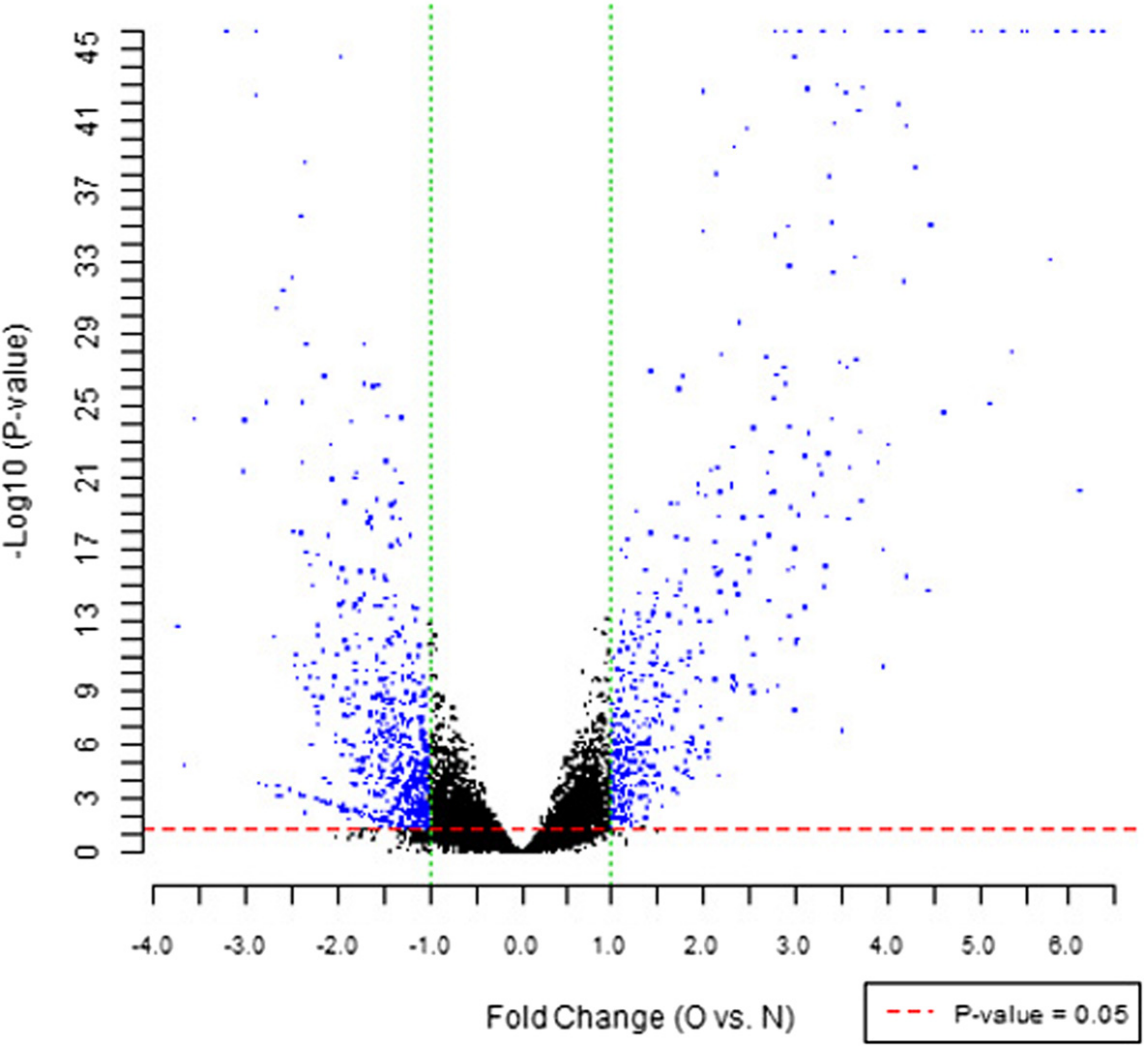
**Volcano plot of distribution of differentially expressed genes between the experimental and control groups.** The dotted line in red and green represent the cut-off, a measurement of gene expression fold-change on the X-axis versus a measure of statistical significance [−Log10 (P-value)] on the Y-axis. Differentially expressed genes are established at |Fold change| ≥1 and P-value < 0.05 (Blue dots in Figure 2).

In order to detect the number of genes affected by CXCR7 transfection, we used gene microarray analysis to compare the fluorescence intensity ratio between the experimental and control group. Logarithm of fluorescence intensity ratio was represented by Fold change, and log2 ratios ≥ 1.0 or log2 ratios ≤ −1.0 means two Fold change (Figure 1). Standard selection criteria to further identify differentially expressed genes are as follows: |log2 ratios| ≥ 1 and P < 0.05 (log2 ratios ≥ 1.0 means up-regulated and log2 ratios ≤ −1.0 represents down-regulated) (Figure 2). Our data indicated CXCR7 transfection up-regulated 529 genes and down-regulated 620 genes.

### Hierarchical clustering analysis showed that CXCR7 over-expression modified gene clusters in PTC cells

**Figure 3:**
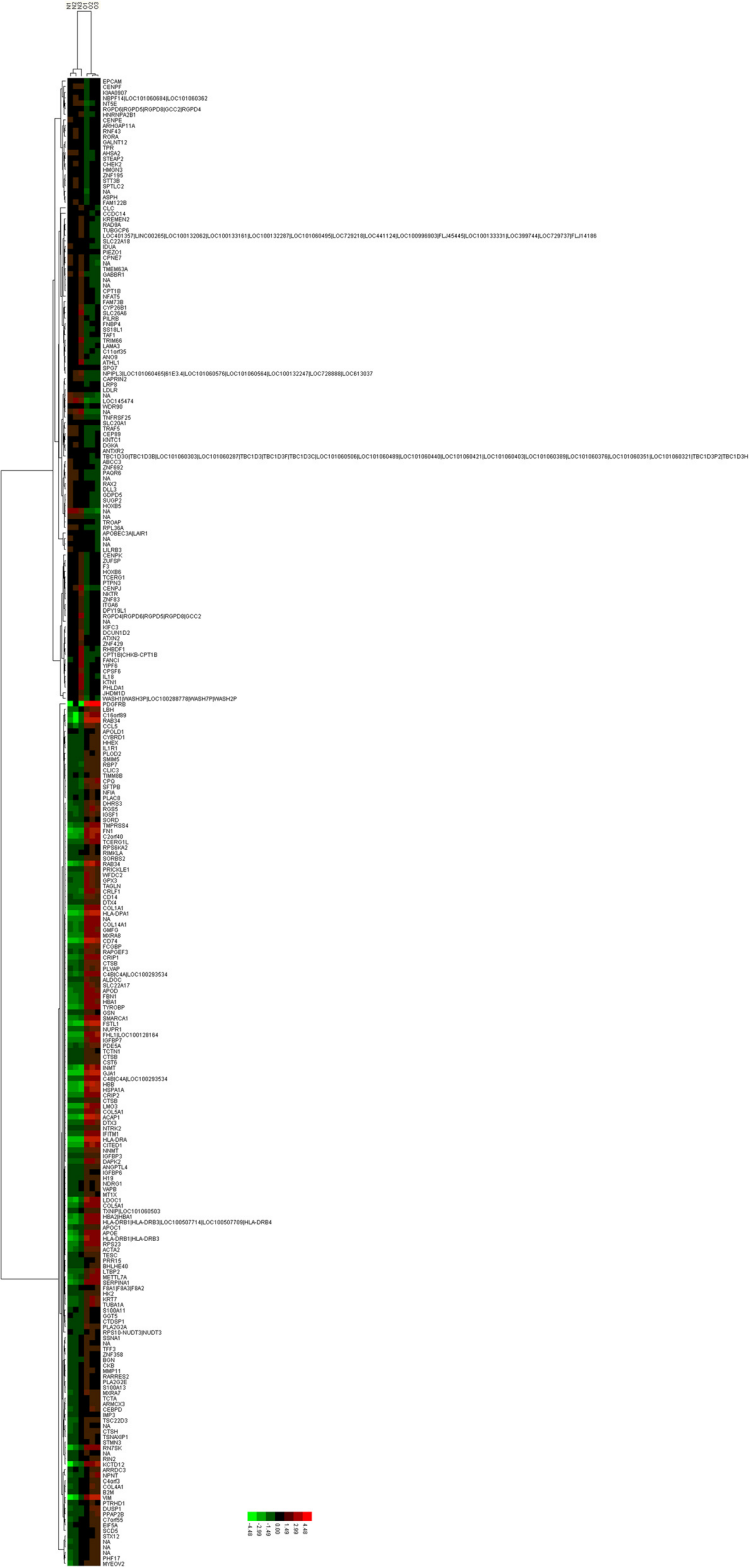
**Hierarchical clustering of differentially expressed genes in the experimental and control groups.** A hierarchical clustering tree indicates the gene expression patterns similarity of the 270 genes between the experimental and control groups. The expression levels of the 270 genes are showed by different color lump: red, high (up-regulated); black, medium; and green, low (down-regulated).

The correlation of expression profiles between the experimental and control groups was demonstrated by unsupervised hierarchical clustering analysis tree (Figure 3). A subset of differential genes that showed similar properties was selected for clustering analysis. An intensity filter was used to select genes where the difference between the maximum and minimum intensity values exceeds 1200 among all microarrays. For this microarray project, the number of genes clustered was 270, among which 156 genes were up-regulated and 114 were down-regulated.

### Pathway analysis showed that genes regulated by CXCR7 over-expression are mainly involved in KEGG signaling pathway

By Pathway analysis, We found that differentially expressed genes were mainly involved in twenty-five KEGG (Kyoto Encyclopedia of Genes and Genomes) classical pathway, especially in ECM-receptor interaction (13 genes), Focal adhesion (18 genes), MAPK signaling pathway (20 genes), Cytokine-cytokine receptor interaction (19 genes), and four BioCarta pathway. Table 2 lists the top 10 significant enrichment pathway terms. And Table 3 lists the top up- and down-regulated genes those were mainly involved in ECM-receptor interaction pathway, Focal adhesion pathway, MAPK signaling pathway and Cytokine-cytokine receptor interaction pathway.

**Table 2:** **The top 10 enrichment pathway terms differentially expressed genes involved in**

**Pathway name**	**Genes count**	**k/K (%)**	**p value**
KEGG_ECM_RECEPTOR_INTERACTION	13	15.48	2.85E-09
KEGG_FOCAL_ADHESION	18	8.96	2.26E-08
KEGG_MAPK_SIGNALING_PATHWAY	20	7.49	7.63E-08
KEGG_CYTOKINE_CYTOKINE_RECEPTOR_INTERACTION	19	7.12	3.54E-07
KEGG_GLYCEROPHOSPHOLIPID_METABOLISM	9	11.69	8.70E-06
KEGG_AXON_GUIDANCE	11	8.53	1.93E-05
BIOCARTA_ATRBRCA_PATHWAY	5	23.81	2.70E-05
KEGG_CELL_ADHESION_MOLECULES_CAMS	11	8.21	2.75E-05
KEGG_COMPLEMENT_AND_COAGULATION_CASCADES	8	11.59	2.92E-05
KEGG_WNT_SIGNALING_PATHWAY	11	7.28	8.21E-05

Note: Genes count is number of genes involved. k/K is the ratio of k and K, where k = number of genes in the overlap between this pathway genes and differentially expressed genes, K = total number of genes in the pathway; P value indicates the significance of Genes count.

**Table 3:** **The top up- and down-regulated genes involved in the significant enrichment pathway**

**Genes**	**Description**	**log2(Ratio) O/N**	**p value (Differentially expressed)**
**KEGG_ECM_RECEPTOR_INTERACTION**			
FN1	Fibronectin 1	5.5287	0
COL1A1	Collagen, type I, alpha 1	4.3820	0
COL5A1	Collagen, type V, alpha 1	3.2131	9.73E-21
COL4A1	Collagen, type IV, alpha 1	2.0595	5.54465E-06
VWF	von Willebrand factor	1.9795	7.64103E-09
LAMA1	Laminin, alpha 1	1.5764	1.77592E-09
TNC	Tenascin C (hexabrachion)	1.0455	6.29032E-07
ITGA6	Integrin, alpha 6	−1.1582	0.001988898
LAMA3	Laminin, alpha 3	−1.5196	1.55E-14
ITGA7	Integrin, alpha 7	−1.9969	8.53527E-12
**KEGG_FOCAL_ADHESION**			
FN1	Fibronectin 1	5.5287	0
COL1A1	Collagen, type I, alpha 1	4.3819	0
PDGFRB	Platelet-derived growth factor receptor, beta polypeptide	3.7321	1.37E-43
COL5A1	Collagen, type V, alpha 1	3.2131	9.73E-21
SHC2	SHC (Src homology 2 domain containing) transforming protein 2	2.1832	2.58E-15
COL4A1	Collagen, type IV, alpha 1	2.0595	5.545E-06
VWF	von Willebrand factor	1.9795	7.641E-09
LAMA3	Laminin, alpha 3	−1.5196	1.55E-14
TNFRSF25	Tumor necrosis factor receptor superfamily, member 25	−1.7049	6.82E-27
ITGA7	Integrin, alpha 7	−1.9969	8.535E-12
**KEGG_MAPK_SIGNALING_PATHWAY**			
PDGFRB	Platelet-derived growth factor receptor, beta	3.7321	1.37E-43
CD14	CD14 molecule	2.7194	7.86E-15
DUSP1	Dual specificity phosphatase 1	2.0457	2.363E-06
PLA2G2A	Phospholipase A2, group IIA (platelets, synovial fluid)	1.9008	5.062E-07
NTRK2	Neurotrophic tyrosine kinase, receptor, type 2	1.8524	1.126E-11
CACNA1I	Calcium channel, voltage-dependent, alpha 1I subunit	1.4363	1.27E-18
ATF4	Activating transcription factor 4 (tax-responsive enhancer element B67)	−1.6707	7.347E-05
PRKACB	Protein kinase, cAMP-dependent, catalytic, beta	−1.7679	2.745E-12
MAP3K4	Mitogen-activated protein kinase kinase kinase 4	−2.1767	0.0014161
NFKB2	Nuclear factor of kappa light polypeptide gene enhancer	−2.2731	1.01E-15
**KEGG_CYTOKINE_CYTOKINE_RECEPTOR_INTERACTION**			
PDGFRB	Platelet-derived growth factor receptor, beta polypeptide	3.7321	1.37E-43
LTB	Lymphotoxin beta (TNF superfamily, member 3)	2.9402	1.67E-33
CXCL12	Chemokine (C-X-C motif) ligand 12 (stromal cell-derived factor 1)	2.7037	5.32E-22
CCL5	Chemokine (C-C motif) ligand 5	2.5225	5.837E-10
CCL21	Chemokine (C-C motif) ligand 21	1.8655	4.374E-07
TNFRSF4	Tumor necrosis factor receptor superfamily, member 4	1.7509	1.69E-15
CXCL2	Chemokine (C-X-C motif) ligand 2	1.4616	3.49E-14
IL23A	Interleukin 23, alpha subunit p19	−1.2503	8.442E-08
ACVR2A	Activin A receptor, type IIA	−1.6469	1.865E-07
TNFRSF25	Tumor necrosis factor receptor superfamily, member 25	−1.7049	6.82E-27

Note: log 2(Ratio) O/N is the logarithm of fluorescence intensity ratio of O and N, where O the experimental group (K1 cell transfected with CXCR7), and N is the control group (K1 cell). |log2 ratios| ≥ 1 and P < 0.05 means the differentially expression genes; P value indicates the significance of log2 (Ratio) O/N.

### Gene ontology analysis results

Gene ontology (GO) database is organized into three categories describing molecular function (MF), biological process (BP), and cellular component (CC). To analyze function differences represented by differentially expressed genes, we conducted a GO analysis. We found, in MF, differentially expressed genes significantly enriched in KINASE_ACTIVITY (29 genes), ION_BINDING (24 genes) and PHOSPHOTRANSFERASE_ACTIVITY_ALCOHOL_GROUP_AS_ACCEPTOR (30 genes); in BP, the top enrichment terms were SIGNAL_TRANSDUCTION (89 genes) and energy metabolism process, such as BIOPOLYMER_METABOLIC_PROCESS (83 genes), CELLULAR_MACROMOLECULE_METABOLIC_PROCESS (68 genes) and PROTEIN_METABOLIC_PROCESS (76 genes); in CC, the genes involved in the CYTOPLASM (113 genes) and cell MEMBRANE_PART (90 genes) expression altered significantly. The top 10 of enrichment terms were showed in Table 4.

**Table 4:** **The top 10 enrichment gene ontology terms**

**Function category**	**Term**	**Genes count**	**k/K (%)**	**p-Value**
**Molecular function**	KINASE_ACTIVITY	29	0.0786	3.03E-11
	ION_BINDING	24	0.0879	1.56E-10
	TRANSFERASE_ACTIVITY_TRANSFERRING_PHOSPHORUS_CONTAINING_GROUPS	30	0.0708	1.81E-10
	CATION_BINDING	19	0.0892	9.88E-09
	ENDOPEPTIDASE_ACTIVITY	14	0.1197	2.15E-08
	PHOSPHOTRANSFERASE_ACTIVITY_ALCOHOL_GROUP_AS_ACCEPTOR	23	0.0689	3.91E-08
	CALCIUM_ION_BINDING	13	0.125	4.04E-08
	HYDROLASE_ACTIVITY_ACTING_ON_ESTER_BONDS	20	0.0743	8.62E-08
	PEPTIDASE_ACTIVITY	17	0.0909	1.10E-07
	PROTEIN_KINASE_ACTIVITY	20	0.0702	2.20E-07
**Biological process**	BIOPOLYMER_METABOLIC_PROCESS	83	0.0493	0.00E?+?00
	SIGNAL_TRANSDUCTION	89	0.0545	0.00E?+?00
	CELLULAR_MACROMOLECULE_METABOLIC_PROCESS	68	0.0592	0.00E?+?00
	CELLULAR_PROTEIN_METABOLIC_PROCESS	68	0.06	0.00E?+?00
	PROTEIN_METABOLIC_PROCESS	76	0.0609	0.00E?+?00
	SYSTEM_DEVELOPMENT	57	0.0662	0.00E?+?00
	MULTICELLULAR_ORGANISMAL_DEVELOPMENT	70	0.0667	0.00E?+?00
	ANATOMICAL_STRUCTURE_DEVELOPMENT	71	0.0701	0.00E?+?00
	RESPONSE_TO_STRESS	39	0.0768	2.52E-14
	ORGAN_DEVELOPMENT	40	0.0701	2.29E-13
**Cellular function**	CYTOPLASM	113	0.0526	0.00E?+?00
	MEMBRANE_PART	90	0.0539	0.00E?+?00
	INTRINSIC_TO_MEMBRANE	76	0.0564	0.00E?+?00
	INTEGRAL_TO_MEMBRANE	76	0.0571	0.00E?+?00
	ORGANELLE_PART	69	0.0576	0.00E?+?00
	INTRACELLULAR_ORGANELLE_PART	69	0.0579	0.00E?+?00
	MEMBRANE	117	0.0587	0.00E?+?00
	NUCLEUS	84	0.0587	0.00E?+?00
	PLASMA_MEMBRANE	86	0.0603	0.00E?+?00
	EXTRACELLULAR_REGION	39	0.0872	3.33E-16

Note: Genes count is number of genes involved. k/K is the ratio of k and K, where k=number of genes in the overlap between this GO term genes and differentially expressed genes, K=total number of genes in the GO term; P value indicates the significance of Genes count.

### Verification of differentially expression genes

**Figure 4:**
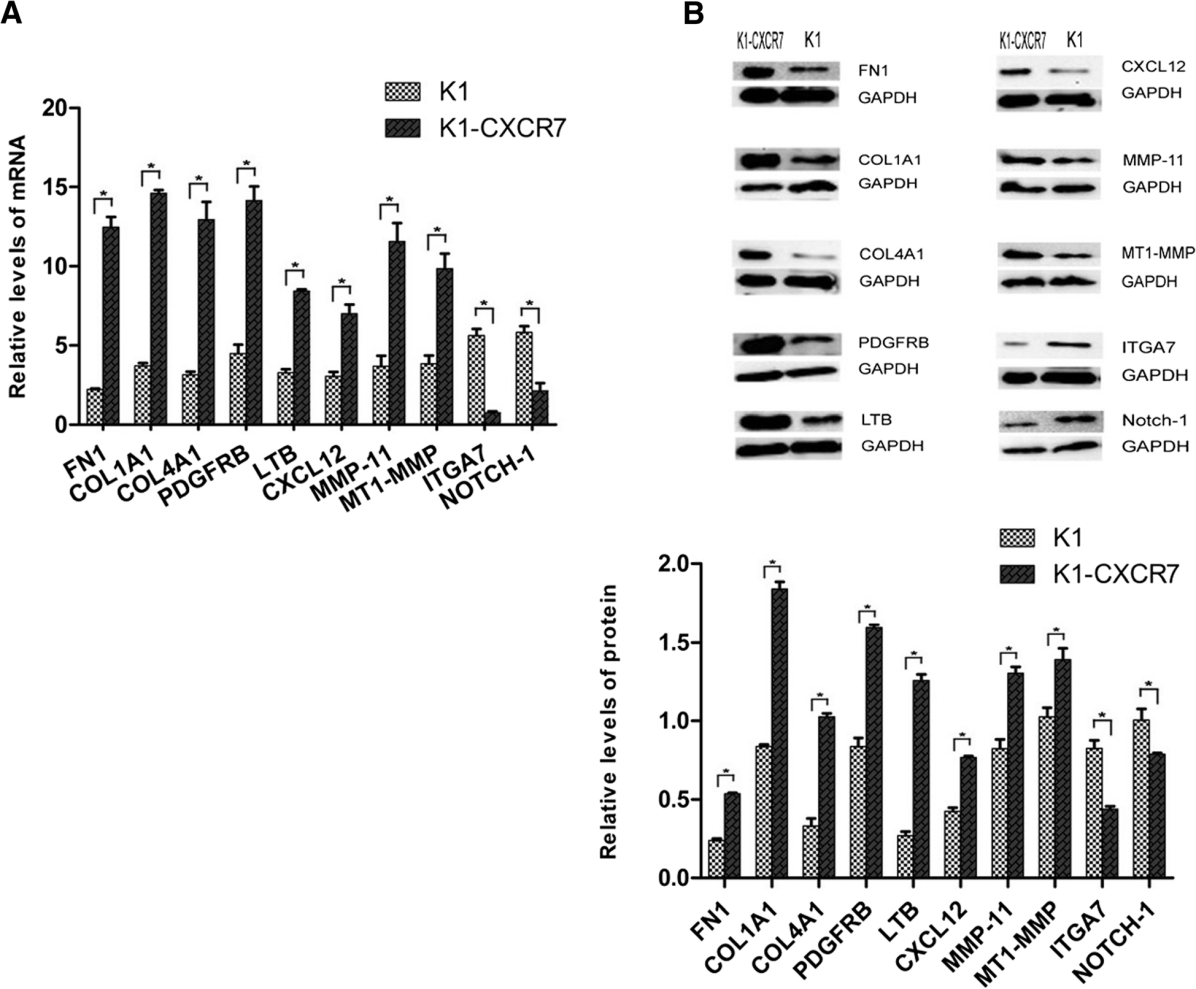
**mRNA and protein expression of differentially expression genes in K1 cell and K1-CXCR7 cell. (A)** Relative mRNA expression level of differentially expression genes (FN1, COL1A1, COL4A1, PDGFRB, LTB, CXCL12, MMP-11, MT1-MMP, ITGA7 and Notch-1). **(B)** Upper panel: protein electrophoregram of differentially expression genes. GAPDH protein was used as a loading control. Lower panel: relative protein levels of differentially expression genes. *p < 0.05.

To partly confirm the expression profiles alteration after PTC cells transfected with CXCR7 gene, we selected up-regulated genes of FN1, COL1A1, COL4A1, PDGFRB, CXCL12, LTB, MMP-11, MT-MMP and down-regulated genes of ITGA7 and Notch-1 for further verification. RT-PCR and Western blot analysis revealed that both mRNA and protein expression levels of genes (FN1, COL1A1,COL4A1, PDGFRB, CXCL12, LTB, MMP-11, MT-MMP) were markedly elevated in K1-CXCR7 cells compared with K1 cells, whereas genes ITGA7 and Notch-1 were the opposite (Figure 4 and Table 5).

**Table 5:** **Expression of mRNA and protein of genes in K1 cells and K1-CXCR7 cells**

**Genes**	**CXCR7 mRNA**	**P value**	**CXCR7 protein**	**P value**
FN1		0.000		0.000
K1	1.772 ± 0.620		0.240 ± 0.010	
K1-CXCR7	12.664 ± 1.080		0.537 ± 0.006	
COL1A1		0.000		0.002
K1	3.404 ± 0.617		0.840 ± 0.010	
K1-CXCR7	14.150 ± 1.014		1.840 ± 0.046	
COL4A1		0.000		0.000
K1	3.146 ± 0.203		0.333 ± 0.045	
K1-CXCR7	12.940 ± 1.111		1.026 ± 0.023	
PDGFRB		0.000		0.000
K1	4.483 ± 0.552		0.839 ± 0.051	
K1-CXCR7	14.441 ± 0.557		1.596 ± 0.019	
LTB		0.000		0.010
K1	3.274 ± 0.219		0.270 ± 0.027	
K1-CXCR7	8.430 ± 0.112		1.257 ± 0.040	
CXCL12		0.000		0.000
K1	3.047 ± 0.276		0.426 ± 0.023	
K1-CXCR7	7.014 ± 0.569		0.770 ± 0.009	
MMP-11		0.000		0.001
K1	3.674 ± 0.674		0.826 ± 0.057	
K1-CXCR7	11.564 ± 1.156		1.304 ± 0.040	
MT1-MMP		0.000		0.003
K1	3.842 ± 0.511		1.026 ± 0.059	
K1-CXCR7	9. 856 ± 0.960		1.392 ± 0.071	
ITGA7		0.000		0.000
K1	5.631 ± 0.413		0.827 ± 0.049	
K1-CXCR7	0.741 ± 0.088		0.439 ± 0.018	
Notch-1		0.000		0.034
K1	5.818 ± 0.395		1.005 ± 0.073	
K1-CXCR7	2.120 ± 0.496		0.789 ± 0.008	

## Discussion

In our study, we filtered 270 differentially expressed genes after PTC cells transfected with CXCR7 cDNA by gene microarray. Among them, 156 genes were up-regulated and 144 genes were down-regulated. The best-known effect of thyroid is an increase in basal energy expenditure achieved by acting on protein, carbohydrate and lipid metabolism. As expected, GO enrichment analysis found several high-enrichment terms linked to metabolism, including biopolymer metabolic process (83 genes), cellular macromolecule metabolic process (68 genes), cellular protein metabolic process (76 genes) and protein metabolic process (76 genes). In addition, differentially expressed genes were significantly involved in signal transduction biological process (89 genes). Pathway enrichment analysis found that the most of differentially expressed genes were enriched in ECM-receptor interaction KEGG pathway (13 genes), focal adhesion KEGG pathway (18 genes), MAPK signaling KEGG pathway (20 genes) and Cytokine-cytokine receptor interaction KEGG pathway (19 genes).

Extracellular matrix (ECM) plays an important role in the development and maintenance of tissue and organ architecture and homeostasis. The synthesis and degradation of ECM components (such as type I collagen, type IV collagen and fibronectin) results in its remodeling and promotes the activated endothelial cells (ECs) proliferation, migration and adhesion to ECM, which contributes to angiogenesis [11].

In this study, fibronectin-1 (FN1) was significantly up-regulated (log2 (Ratio) =5.5288). It was involved in ECM-receptor interaction pathway, focal adhesion pathway, pathways in cancer and regulation of actin cytoskeleton pathway. As an important ECM component, FN1 regulates ECs survival, proliferation, adhesion, migration and angiogenesis [12]. Several researches reported that FN1 was over-expressed in PTC, and it may be a useful biomarker to diagnose PTC [13-15]. ECs facilitate tumor angiogenic process through the activation of focal adhesion kinase (FAK) and downstream PI3K/Akt signal pathway, as well as the activation of NF-κB [12,16]. So, we considered that CXCR7 might promote EC adhesion to ECM by up-regulating the expression of FN1, inducing FAK-mediated activation of PI3K/Akt as well as NF-κB pathway, thereby regulating PTC progression.

COL1A1 (collagen, type I, alpha 1) was also obviously up-regulated (log2 (Ratio) =4.3820), involved in ECM-receptor interaction pathway and focal adhesion pathway in our study, as well as COL4A1 (collagen, type IV, alpha 1). As is well-known, COL1A1 is expressed in many tumor cells and tumor-associated stromal cells. Several researches have demonstrated that COL1A1 plays an important role in angiogenesis and desmoplasia, and the over-expression of COL1A1 was associated with invasion process in PTC [17,18]. What’s more, it was reported that PI3K/AKT and NF-κB pathway were involved in regulating the expression of COL1A1 [19,20]. And COL4A1, as an essential component of ECM, plays an important role in angiogenesis and tumor progression [21]. These suggested that PI3K/AKT and NF-κB pathway may mediate the induction of collagen (COL1A1 and COL4A1) by CXCR7 in PTC cells.

Chemokines and their receptors are best known for their ability to mediate the direct migration of leukocytes in the immune system. As we all known, a close connection exists between inflammation and cancer. Recently, accumulating evidence suggests that alterations in immune function may play a crucial role in PTC initiation. And a history of autoimmune disease, always resulting in tissue destruction and inflammation, has also been associated with the increased risk of PTC [22-24]. As shown in our study, several up-regulated differentially expressed genes were significantly involved in Cytokine-cytokine receptor interaction pathway, including PDGFRB (log2 (Ratio) =3.7321), LTB (log2 (Ratio) =2.9402), CXCL12 (log2 (Ratio) =2.7037). They are all important pro-inflammatory molecules, which play important roles in regulating tumor cells proliferation, invasion, metastasis, angiogenesis and apoptosis. Platelet-derived growth factors (PDGFs), a family of peptides of growth factors, bind to their receptors (PDGFR-α and -β) and stimulate growth and diffusion of cancer cells. It was reported that PDGFR-α and -β were expressed in PTC but not found in normal thyroid tissues [25]. And PDGFR-β can induce the transcription and secretion of vascular endothelial growth factor (VEGF), which plays a critical role in tumor growth, angiogenesis and metastasis [26]. LTB, lymphotoxin beta (TNF superfamily, member 3), is a multifunctional pro-inflammatory cytokine that belongs to the tumor necrosis factor (TNF) superfamily. As we all known, TNF is involved in the regulation of multiple biological processes, including cell proliferation, differentiation and apoptosis. It was reported that an autocrine inflammatory cytokine network of inflammatory cytokine TNF-α, angiogenic factor VEGF, and chemokine CXCL12 existed in ovarian cancer microenvironment and stimulated tumor neoangiogenesis [27]. What’s more, TNF-αinduced the production of VEGF and CXCL12, and VEGF also induced CXCL12 [28,29]. Meanwhile, CXCL12 and VEGF were synergized in facilitating angiogenesis of ovarian cancer [30]. In addition, anti-apoptotic and pro-inflammatory abilities of TNF-αwere resulted from the induction of NF-κB [31,32]. Indeed, NF-κB is one of the main TNF-α downstream effectors, and functions as a transcription factor to regulate genes associated with inflammatory, anti-apoptotic and cell proliferation. Madge LA et al. [33] also demonstrated that lymphotoxin-beta receptor (LTβR) ligands LIGHT and LTα1β2 activate NF-κB pathway, and then up-regulated chemokine CXCL12. CXCL12, also named stromal cell-derived factor 1 (SDF-1), functions as a chemotactic factor for many cells, including T cells, pre-B-cells, monocytes, dendritic cells, and hematopoietic progenitor cells [34,35]. It was also involved in tumor cells migration, invasion and metastasis [36,37]. What’s important, we have demonstrated a significantly higher expression level of SDF-1 in PTC tissue compared with peritumoral nonmalignant tissue and thyroid benign lesion tissue, and the expression of SDF-1 was closely associated with lymph node metastasis of PTC [8]. In addition, our research results suggested that SDF-1 binding to its receptor CXCR7 regulated the directed migration and invasion of PTC cells [9]. So, it is likely that CXCR7 interaction with its ligand CXCL12, induced expression alterations of pro-inflammation genes through the induction of NF-κB signaling, therefore regulated anti-apoptotic and proliferation of PTC cells.

Matrix metalloproteinases (MMPs) mediate tumor cell invasion and metastasis by degrading of ECM. Maeta H et al. [38] demonstrated that the expression of MMPs (MMP-2 and MMP-9), as well as their inhibitors (TIMP-1 and TIMP-2) were enhanced in aggressive PTC. Wani N et al. [39] reported that CXCR7 promoted the metastasis of breast cancer by up-regulating the activity of MMPs. In our study, MMP-11 and membrane type 1 matrix metalloproteinase (MT1-MMP) were both up-regulated (log2 (Ratio) =1.5055, log2 (Ratio) =1.0861), which demonstrated that CXCR7 might promote the secretion of MMP-11 and MT1-MMP by up-regulating genes expression at transcriptional level. Although MMP-11 can’t degrade any ECM component, it is also associated with tumor progression and poor prognosis. MMP-11 was found negative in normal thyroid tissue and thyroid follicular cells but significantly expressed in PTC tissues and thyroid carcinoma cell lines [18,40]. Focal degradation of ECM is the key step in the invasion of cancer cells, MT1-MMP degrades ECM by activating proMMP-2, which promote cancer cells invasion [41]. Nakamura H et al. [42] reported that MT1-MMP expression was correlated with the activation of proMMP-2 and lymph node metastasis of PTC, which suggested expression of proMMP-2 and MT1-MMP-mediated activation played a vital role in the lymph node metastasis of PTC. These results indicated that chemokine receptor CXCR7-induced the transcriptional activation of MMPs promoted the secretion of MMPs proteins and resulted in the degradation of ECM, which induced PTC invasion and lymph node metastasis.

In addition, ITGA7 (integrin, alpha 7), as a cell adhesion molecule, contributes to the interaction between ECM and cells by binding to its ligand integrin β and is involved in multiple biological processes, such as human tissue development, tissue differentiation, and immune responses. Recently, increasing evidences have demonstrated ITGA7 as a tumor suppressor gene in many human malignant neoplasms [43-45], including prostate cancer, liver cancer, glioblastoma multiforme and leiomyosarcoma. Han YC et al. [44] reported that integrin-link kinase interaction with miniature chromosome maintenance 7 (MCM7) and MCM7 phosphorylation may be a critical event in ITGA7 signaling pathway leading to tumor suppression. Tan LZ et al. [45] reported a possible mechanisms of ITGA7-mediated tumor cell growth suppression is that IGTA7 interacts with tissue inhibitor of metalloproteinase 3 (TIMP3), results in the relocation of NF-κB from nucleus to cytoplasm, and down-regulates cyclin D1. These processes led to a remarkable suppression of cell growth. In our study, IGTA7 gene was also significantly down-regulated (log2 (Ratio) = −1.99689) in K1-CXCR7 cell, which suggested that CXCR7 might promote the progression of PTC cell by inhibiting the expression of IGTA7, but functions of ITGA7 in PTC need to be further studied.

Notch1, as a multifunction transmembrane receptor, plays a key role in metazoan development. It also participates in the maintenance of tissue homeostasis by regulating cell proliferation, differentiation and apoptosis [46,47]. Notch1 functions as either a tumor suppressor gene or an oncogene in many human carcinomas, which is cell type-specific. In thyroid cancers, the role of Notch1 signaling is tumor histological differentiation dependent. Notch1 signaling was significantly down-regulated in human anaplastic thyroid carcinoma compared with normal thyroid cells, over-expression of Notch1 reduced cancer cells growth and restored differentiation [48]. It was reported that Notch1 acted as a tumor suppressor in medullary thyroid carcinoma (MTC), and activation of Notch1 inhibited growth of MTC cells and induced apoptosis of MTC cells [49-51]. However, Notch1 as an oncogene or a tumor suppressor gene in PTC remains controversial. Zhang J et al. [52] showed that Notch1 expression was higher in PTC compared with normal thyroid tissues, and higher expression levels of Notch1 was closely associated with lymphatic metastasis and poor prognosis of PTC [53,54]. However, Xiao X et al. [55] showed that Notch1 expression was minimal in papillary and follicular thyroid cancer cells, and activation of Notch1 inhibited growth and proliferation of thyroid cancer cells. Our data indicated that Notch-1 gene was significantly down-regulated (log2 (Ratio) = −1.3895) in K1-CXCR7 cell, which suggested Notch-1 gene might have the effect of tumor suppression in PTC. Several studies have shown that Notch1 signaling might be involved in regulating tumor progression through interacting with multiple signaling pathways, including PI3K/AKT, NF-κB and Wnt pathway [56-58], but further studies were needed to identify the functions and mechanisms of Notch1 signaling in PTC.

Other than the genes mentioned above, there are some genes found to be regulated by CXCR7 in our study but have few reports on, such as deltex homolog (DTX3), matrix-remodeling associated 7 (MXRA7). In our study, expressions of DTX3 and MXRA7 were dramatically increased at transcriptional level (log2 (Ratio) =3.2667, log2 (Ratio) =2.3092). It was reported that DTX3 may function as an ubiquitin ligase protein, which regulate the Notch pathway via some ubiquitin ligase activity [59]. Veiga-Castelli LC et al. [60] demonstrated MXRA7 was over-expressed in ectopic endometrium, and it may result in tissue remodeling according to Gene Ontology analysis. However, the exact functions of these genes are not clear. Further studies on these genes are needed to demonstrate their function and correlation with PTC progression.

## Conclusions

In summary, here we demonstrate that CXCR7 mediates the transcriptional expression of multiple signaling molecules, including FN1, COL1A1, COL4A1, PDGFRB, MMP-11, MT1-MMP, LTB, CXCL12, ITGA7 and Notch-1. These signal molecules are involved in PI3K/AKT, NF-κB, Notch signal pathway, which may be associated with papillary thyroid carcinoma growth and metastasis. Chemokine receptor CXCR7 may promote PTC growth and metastasis via the activation of PI3K/AKT pathway and its downstream NF-κB signaling, as well as the down-regulation of Notch1 signaling. In addition, several genes with unknown function were found by gene microarray, such as DTX3 and MXRA7, which need to be further studied.
